# Prospects and applications of on-chip lasers

**DOI:** 10.1186/s43593-022-00027-x

**Published:** 2023-01-04

**Authors:** Zhican Zhou, Xiangpeng Ou, Yuetong Fang, Emad Alkhazraji, Renjing Xu, Yating Wan, John E. Bowers

**Affiliations:** 1 Integrated Photonics Lab, King Abdullah University of Science and Technology, Thuwal, Makkah Province Saudi Arabia; 2 Function Hub, The Hong Kong University of Science and Technology (Guangzhou), Guangdong, China; 3 Institute for Energy Efficiency, University of California, Santa Barbara, Santa Barbara, CA 93106 USA

**Keywords:** On-chip lasers, Silicon photonics, Photonic integration, Communication, LiDAR, Optical computing

## Abstract

Integrated silicon photonics has sparked a significant ramp-up of investment in both academia and industry as a scalable, power-efficient, and eco-friendly solution. At the heart of this platform is the light source, which in itself, has been the focus of research and development extensively. This paper sheds light and conveys our perspective on the current state-of-the-art in different aspects of application-driven on-chip silicon lasers. We tackle this from two perspectives: device-level and system-wide points of view. In the former, the different routes taken in integrating on-chip lasers are explored from different material systems to the chosen integration methodologies. Then, the discussion focus is shifted towards system-wide applications that show great prospects in incorporating photonic integrated circuits (PIC) with on-chip lasers and active devices, namely, optical communications and interconnects, optical phased array-based LiDAR, sensors for chemical and biological analysis, integrated quantum technologies, and finally, optical computing. By leveraging the myriad inherent attractive features of integrated silicon photonics, this paper aims to inspire further development in incorporating PICs with on-chip lasers in, but not limited to, these applications for substantial performance gains, green solutions, and mass production.

## Introduction

The global consumer Internet traffic grew fourfold from 2017 to 2022 with a compound annual growth rate of 31% and will reach 4.8 Zettabytes (ZB) per year by 2022, or 396 Exabytes (EB) per month [1]. If you imagine every one of those gigabytes as a brick, 4.8 trillion of them would be able to build more than a thousand Great Walls in China. Faced with this ever-growing demand to expand the bandwidth and reduce the power consumption, photonic integrated circuits (PICs) are proposed to catalyze powerful new technologies, much like electronic circuits have in the past, with distinct advantages in scalable capacity, transparency, low latency, and low energy per bit [2].

Numerous optical-related technologies are emerging in control, intelligent computing, information security [3, 4], etc. To date, optical communication has comprised the backbone of telecommunication applications and is replacing electrical interconnect links at increasingly short lengths. To enable an optical network-on-chip (NoC) for communication between multiple cores and eventually for on-chip communications, robust operation is required to sustain high temperatures near the electronic processors, and volume manufacturability and overall system cost need to be technically and economically competitive. Bandwidth density, energy efficiency, and latency are the key parameters, and projections are that the system energy target will be ∼100 fJ/bit, with about 10–20 fJ/bit allocated for the optical source [5]. Nevertheless, designing photonic devices that meet all these criteria is challenging, and integration of as many components as possible on a single chip increases affordability and process compatibility.

In a transition from assembled optics relegated to bulky, bench-scale apparatuses to on-chip photonics with compact form factors, there are primarily two aspects: III–V-based PICs and silicon photonics. With the property of direct bandgap and the linear electro-optic (Pockels) effect, III–V-based PICs have been commercialized in the last half century and provide innovative industry-leading connectivity solutions including high-end subcomponent technology, and systems for network infrastructure. [6] However, a pure III–V approach limits the foundry to the use of relatively small wafers, which is ~ 150 mm in diameter for indium phosphide (InP) and ~ 200 mm for gallium arsenide (GaAs). The waveguide loss in GaAs or InP waveguides is typically over 1 dB/cm, which greatly hinders performance in many applications. Furthermore, the inherently small index contrast in the III–V stacks forbids the formation of tight bends and high-confinement waveguides for high-density integration.

Alternatively, silicon becomes an obvious choice due to its superior optical properties and its compatibility with the mature complementary metal–oxide–semiconductor (CMOS) technology. Moreover, the large contrast between silicon and silicon dioxide allows photonic devices to be fabricated in a compact size with a loss on the order of 0.1 dB/cm to 1 dB/cm [7]. The range of optical components demonstrated on the silicon-on-insulator (SOI) platform is extremely impressive, including not only passive devices like low-loss waveguides, high-*Q* resonators, polarization control devices, couplers, multiplexers, filters, etc., but also active high-bandwidth modulators based on silicon *pn* junctions [8], and high-speed photodetectors (PDs) based on epitaxially grown germanium films [9]. Nevertheless, as an indirect bandgap material, silicon has a low emission efficiency. Therefore, on-chip light sources lagged far behind other silicon-based photonic devices and became a bottleneck for mass production. To break such a barrier, a great deal of research has emerged in the field of on-chip light sources in the past decades. The requirements of ideal integrated on-chip lasers have been identified by academia and the industry to meet different criteria [10, 11]. In terms of operation performance, they should generate sufficiently large power with high power efficiency to support interconnects at a compact scale. They need also to be electrically-driven (pumped) lasers, to benefit from the CMOS electronic environment and for compactness and high integration density. They should also lase in Continuous-wave (CW) emission mode, in order to avoid any capacitive transient processes, while withstanding high operation temperatures close to CMOS electronic processing units (30 to 150 °C). From a spectral point of view, it is desirable that the integrated on-chip lasers emit in the commonly used fiber-based communications, viz*.,* O-band (1310 nm) and C-band (1550 nm) for seamless interconnectivity with optical fiber networks. Moreover, they should suffer from as low phase/frequency noise as possible to achieve narrow linewidth (high monochromaticity) emission spectra, which is paramount in several applications, and to maximize their temporal and spatial coherence. Lastly, the integrated on-chip lasers need to be compatible with silicon-based CMOS processes for large-scale manufacturing and scalability. To that end, from a physical standpoint, they must have a minimal footprint for the sake of co-integration with CMOS technology whose footprint remains to be orders of magnitude less than that of photonics. In addition, as a core part of PICs, integrated on-chip lasers must possess a high degree of flexibility, robustness, and reliability.

**Fig. 1:**
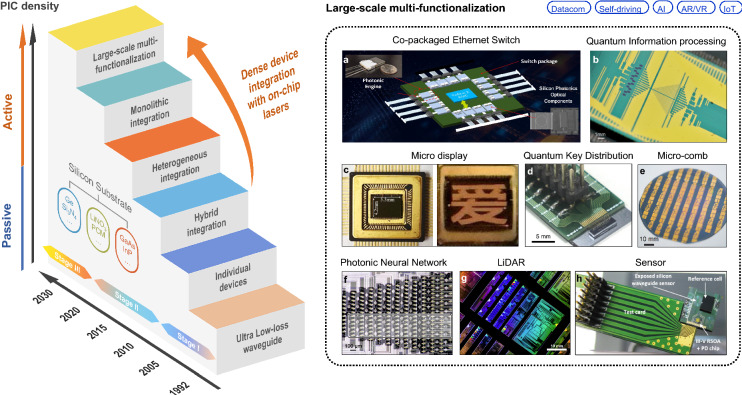
**Progress of silicon-based photonic integration with different development stages since 1992**. Examples of Large-scale multi-functionalization are listed: **a** Co-packaged Ethernet Switch. Image credit: Intel [17]. **b** Quantum Information processing. Adapted with permission from [18], copyright 2022, AAAS. **c** Micro display. Adapted with permission from [19], copyright 2021, IEEE. **d** Quantum Key Distribution (QKD). Adapted with permission from [20], copyright 2019, NPG. **e** Micro-comb. Adapted with permission from [21], copyright 2021, AAAS. **f** Photonic Neural Network Adapted with permission from [22], copyright 2017, NPG. *g* LiDAR Image credit: MIT Lincoln Laboratory [23]. **h** Sensor. Adapted with permission from [24], copyright 2019, SPIE

Excellent reviews of recent advances and future perspectives can be found in [12–16]. In this paper, we further discuss the prospects of on-chip lasers from the perspective of incorporating cutting-edge applications. Hence in what follows, we will focus on the on-chip integration of the key element in PIC: a compact, energy-efficient, and robust laser source. We will start with an overview of the decades of efforts in exploring new materials and integration technologies for on-chip lasers, then analyze the corresponding material system compatibility and efficient channeling of energy from device to device on-chip. In Fig. 1, we summarize the silicon-based photonic integration development stages with several representative works. In the next few sections, we discuss the prospects and demonstrated application scenarios from communications to LiDAR, point-of-care biosensors, quantum computing and communication, and to optical computing.

## IV-based silicon laser

**Fig. 2:**
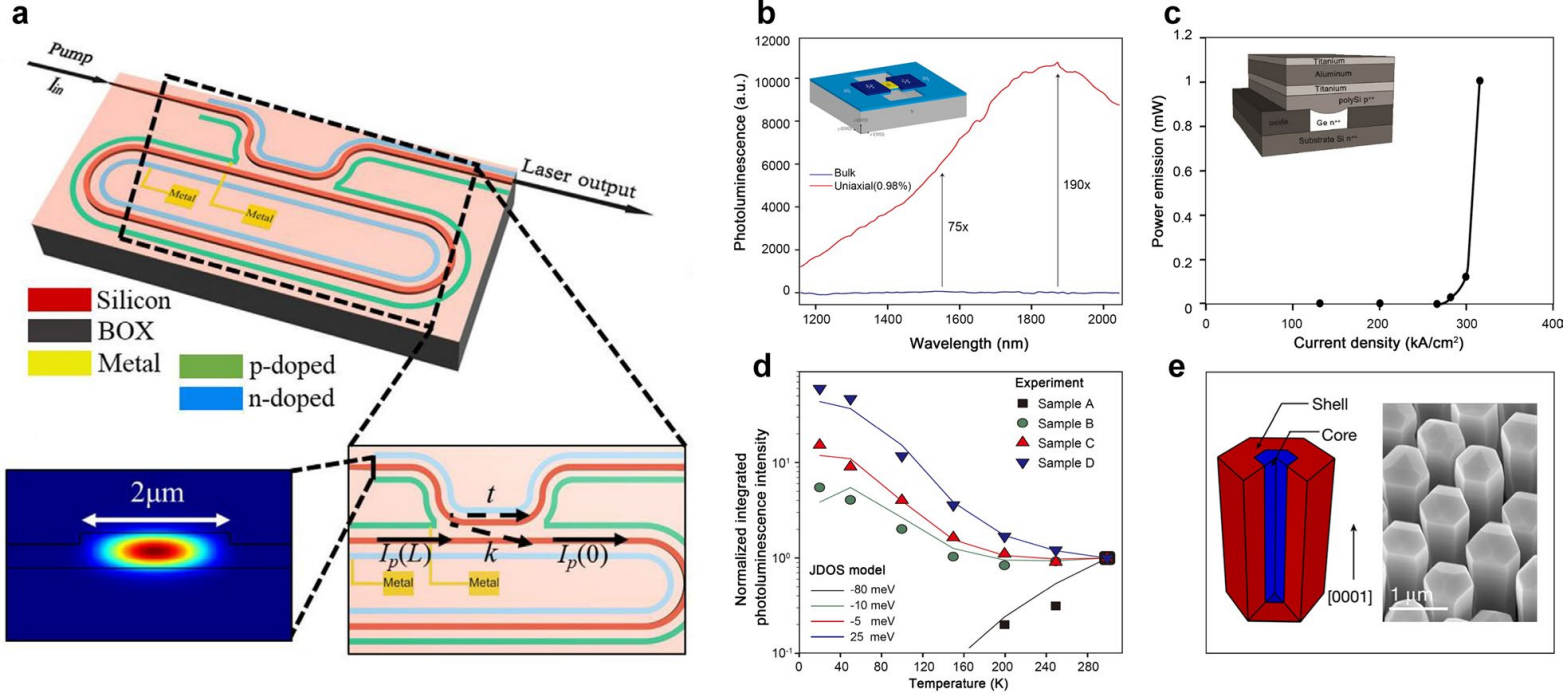
**Concepts for on-chip silicon-based lasers using group-IV materials**. **a** Silicon Raman racetrack ring laser Adapted with permission from [31], copyright 2022, WILEY. **b** Micromachining-based GOI structure realizes direct-bandgap light-emitting under tensile strain. Adapted with permission from [37], copyright 2012, NPG. **c** The first electrically pumped Ge laser at room temperature (combination of tensile strain and n-type doping). Adapted with permission from [38], copyright 2012, OPG. **d** Lasing in a direct-bandgap group IV system created by alloying GeSn without mechanically introducing strain. Adapted with permission from [40], copyright 2012, AIP. **e** Light emission from direct-bandgap hexagonal Ge and SiGe alloys. Adapted with permission from [26], copyright 2020, NPG

To achieve efficient light emission in Group-IV materials, the initial focus was on modifying silicon from a material engineering perspective with porous silicon [25], hexagonal silicon [26], silicon quantum dots (QDs) [27, 28], etc. Since silicon has a high Raman coefficient, silicon-based Raman lasers have the potential to achieve sufficiently large gains [29, 30]. In 2007, Intel demonstrated an optically pumped silicon Raman laser based on a racetrack ring. CW light emission was achieved with a lasing threshold of 20 mW, a slope efficiency of 28%, an output power of 50 mW, and a SMSR of > 80 dB [29]. Parallel to the experimental demonstrations, Raman lasing in high-*Q* multimode cavities has also been investigated theoretically (Fig. 2a) [31]. However, there is no clear roadmap yet for an electrically injected silicon Raman laser. Meanwhile, since the energy difference (136 meV) between the direct valley (Γ) and indirect valleys (L) in Ge is small, high emission efficiency can be achieved at its direct bandgap energy near 0.8 eV (1550 nm) through the appropriate band structure engineering [32, 33], including *n*-type doping, tensile strain introduction, and germanium-tin (GeSn) alloy.

In *n*-doped Ge, the indirect L valley is filled with extrinsic electrons, which raised the Fermi level to the minimum of the direct Γ valley, thus increasing the injected electron density in the Γ valley. Sun et al. theoretically calculated the net gain versus injected excess carrier density. It is proposed that with a low tensile strain (*e.g.*, 0.25%), *n*-type doping is essential to obtain direct-gap net gain in Ge. At an active doping concentration of 7 × 10^19^ cm^−3^, a net gain of 500 cm^−1^ can be realized [34].

For the tensile strain approach, as the tensile strain increases, the direct bandgap shrinks faster than the indirect bandgap in Ge, resulting in a reduced energy difference between the direct Γ valley and the indirect L valley. The calculated results, based on linear deformation potential theory, indicated that Ge would be converted into a direct bandgap material at a 2% biaxial tensile strain or 4.6% uniaxial tensile strain [35, 36]. So far, several methods have been investigated to introduce large and flexible tensile strain in Ge. In 2012, Jain et al. reported a micromachine-based Ge energy band tuning technology [37], where suspended Si_3_N_4_ and Ge-on-Insulator (GOI) structure was employed to introduce certain controllable tensile strain into Ge to transform its band structure into a direct bandgap (Fig. 2b). This was demonstrated by photoluminescence (PL) measurements where optical emission of the strained structure has an emission enhancement over bulk germanium by more than 130 times at 1550 nm. Later, using a combination of ~ 2% tensile strain and 4 × 10^19^ cm^−3^ *n*-type doping, Camacho-Aguilera et al*.* demonstrated the first electrically pumped Ge laser with an output power > 1 mW and a gain spectrum > 200 nm at room temperature, as shown in Fig. 2c [38]. However, this electrically pumped Ge laser has a relatively high lasing threshold of 280 kA/cm^2^ and low emission efficiency.

For the GeSn technique, when the fraction of Sn increased to 6.5%, GeSn alloy becomes a direct bandgap material [39]. The main obstacle to this approach, though, is the large mismatch lattice between Sn and Ge and the low solubility (< 1%) of Sn in Ge. Molecular beam epitaxial (MBE) and chemical vapor deposition (CVD) were later introduced to address these issues. In 2015, Wirths et al. first realized an optically pumped GeSn alloy laser operating at a low temperature ≤ 90 K (Fig. 2d) [40]. In 2020, the first electrically pumped GeSn laser was demonstrated based on a GeSn/SiGeSn double-heterostructure with a narrow light linewidth of 0.13 nm, a maximum lasing temperature of 100 K, and a 2300 nm peak wavelength [41]. In a parallel effort, hexagonal Ge and SiGe alloys have been intensively explored to achieve efficient light emission. As shown in Fig. 2e [26]. Fadaly et al. demonstrated that compositional engineering could convert hex-Si_1-*x*_Ge_*x*_ to direct bandgap, which has a widely tunable emission wavelength and a high emission yield comparable to direct-bandgap III–V semiconductors.

In summary, many efforts have been explored to achieve on-chip light sources based on group-IV materials which include Ge, silicon, and their alloys. With the support of the developed CMOS infrastructure, these initiatives aim to create a monolithically integrated laser for silicon photonics. The demonstrations of electrically pumped Ge laser, GeSn laser, and optical pumped hexagonal SiGe laser represent major technological leaps to realize an on-chip light source on the IV platform. Nevertheless, the threshold current and luminous efficiency of these solutions fall far behind the commercially relevant performance levels achieved regularly in the III–V systems. Ge-based lasers should still be carefully studied and optimized before they will be used in practical applications.

## III–V-based silicon laser

III–V materials are direct bandgap semiconductors with a substantially higher emission efficiency than indirect bandgap materials. Multiple strategies have been explored to integrate III–V lasers tightly onto the silicon substrates, including hybrid integration, heterogeneous integration based on wafer bonding, and monolithic integration based on direct epitaxial growth. Table 1 remarks an intuitive comparison of these commonly used integration technologies. Here in this section, we will first state the differences between hybrid and heterogeneous integration. Subsequently, a comparison of three typical bonding techniques for heterogeneous integration will be presented, along with an insight into the research progress and commercial successes achieved by heterogeneous integration. We then proceed to another important technology, monolithic integration. In the beginning, we summarized the process development of monolithic integration, and then we introduce the current scheme and results of coupling the epitaxially grown III–V lasers to the waveguides.

**Table 1:** Comparison of different III–V/Si Integration technologies

III–V/Si Integration technologies		Thermal conductivity	Surface requirement	Substrate material required	Active–passive coupling loss	Assemble cost	Test complexity
Hybrid		N/A	N/A	III–V & SOI	2–8 dB [42]	External laser packaging and coupling	High: die & wafer level
Heterogenous	BCB	Low	Low	III–V & SOI	0.2–0.5 dB [21, 43]	N/A	Low: wafer level
	Metal	High	Low				
	Direct	High	High				
Monolithic		High	High	Silicon	Preliminary result:-7.35 dB [44] Assuming butt-joint regrowth: 0.1–0.5 dB	N/A	Low: wafer level

### Hybrid and heterogeneous integration

**Fig. 3:**
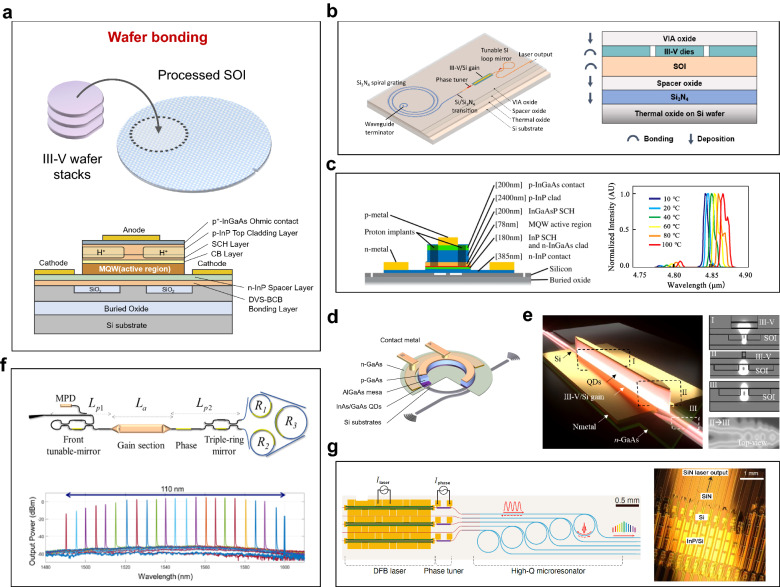
**Heterogeneous integration technology**. **a** A schematic illustration of the bonding process. **b** A fully integrated Si3N4-based laser through multiple wafer bonding. Adapted with permission from [58], copyright 2020, OPG. **c** A heterogeneously-integrated QCL with the longest wavelength at 4.8 μm. Adapted with permission from [63], copyright 2016, OPG. **d** Compact low-threshold micro-ring lasers with heterogeneously integrated QD epitaxial material as an active layer. Adapted with permission from [65], copyright 2019, OPG. **e** Heterogeneously integrated QD-DFB lasers with efficient light coupling to the silicon waveguide. Adapted with permission from [66], copyright 2021, WILEY. **f** Widely tunable lasers using a coupled-triple ring structure. Adapted with permission from [69], copyright 2019, IEEE. **g** Laser soliton microcombs. Adapted with permission from [21], copyright 2021, AAAS

Hybrid and heterogeneous integration both refer to integrated process technologies of different materials in microelectronics and photonics, but there are some distinctive differences between the two concepts. *Hybrid integration* is an integration process that connects multiple fully processed dies into one single package at the final packaging stage, which has the advantage of being able to test and characterize devices and discard non-functional components before the integration. *Heterogeneous integration*, on the other hand, bonds unpatterned III–V thin films to silicon or Si_3_N_4_ wafers at the early- to mid-stages with a coarse alignment, and then define devices lithographically on the full wafer scale. A schematic illustration of the heterogeneous bonding process is shown in Fig. 3a. This process technology shifts the manufacturing of on-chip lasers from chip-level to automatic wafer-level, and eliminates the requirements of active alignment between integrated elements, thereby enhancing integration density, production volume, and cost-effectiveness.

Currently, the most widely adopted wafer-bonding methods include polymer bonding, metal bonding, and direct bonding based on interfacial materials. *The polymer bonding* method employs polymers, such as benzocyclobutene (BCB) and SU-8, in the bonding interface as an adhesive layer. Polymer bonding has advantages in terms of tolerance of roughness and cleanliness of the wafer surface, bonding strength, thermal stability, and cost efficiency. Since its first demonstration back in 2006 [45], polymer bonding-based laser performance has been improved with a waveguide-coupled single-facet output power of 14 mW, a side-mode-suppression-ratio (SMSR) above 50 dB for a distributed feedback (DFB) laser [46]. Nevertheless, the thermal conductivity of polymers is a problem, which can cause poor heat dissipation in bonded devices unless the bonding layer is less than 100 nm thick. *Metal bonding* is another attractive technology to achieve intermediate layer bonding. Compared to BCB bonding, metal bonding provides a low resistance Ohmic contact and high thermal conductivity, which is beneficial for thermal management [47]. However, the metal intermediate layer leads to high light absorption and the risk of metal contamination. The former causes large optical loss in the PICs and degrades the evanescent coupling efficiency, whereas the latter restricts metal bonding to back-end processing only and deteriorates its full compatibility with CMOS processing. These two factors constrain the use of the metal bonding method mainly to the packaging stage.

To date, direct bonding is the most commonly used bonding technique, where two material surfaces are joined together through a strong interfacial bond without the use of adhesion layers. After a series of surface treatments (polishing, flattening, plasma cleaning, etc.), the III–V materials can be bonded directly to the SOI wafer through intermolecular forces, with a strong bonding strength and a high yield [48]. In 2006, UCSB demonstrated the first electrically pumped silicon-based heterogeneous integrated laser using direct bonding [49]. By utilizing III–V materials for optical gain/modulation and silicon for optical waveguiding, various kinds of heterogeneously integrated lasers, including DFB [50], distributed Bragg reflector (DBR) [51], and sampled-grating DBR (SG-DBR) [52], were realized with high performance exceeding what can be achieved in native substrates. Ten years after demonstrating this technology, Intel introduced its own family of 100 Gb/s transceivers based on heterogeneously integrated lasers. Ever since then, a total of over 6 million 100-gigabit optical modules with heterogeneously-integrated on-chip lasers have been shipped, generating over $1 billion of revenue [53].

The bonding techniques can accommodate not only III–V materials for gain with versatile spectral coverage, but also a variety of exotic materials, such as LiNO_3_ [54], Ce: YIG [55], phase-change materials (PCM) [56], and two-dimensional materials [57], for high-performance modulation, nonlinearities, magnetic properties, etc. In addition, multilayer structures can be created through multiple wafer bonding. Index mismatch between different material stacks can thus be bridged through intermediate layers. For example, lasing in a fully integrated Si_3_N_4_-based external cavity was realized for the first time using an intermediate silicon layer [58]. The multilayer III–V/silicon/Si_3_N_4_ structure (Fig. 3b) allows the implementation of Si_3_N_4_ with the III–V gain, which works well at datacom and telecom wavelengths (1.2–1.6 µm). Operation at shorter wavelengths is possible with the development of efficient III–V/Si_3_N_4_ transitions, opening up the visible window and allowing one PIC to operate from 0.4 µm to 4 µm [59, 60]. In addition to extending the operating wavelength of silicon-based PICs to visible light, enhanced performance of devices with ultra-low propagation loss, low-temperature insensitivity, and high power-handling capability are possible. Due to the low thermo-optic coefficient in Si_3_N_4_ and SiO_2_, the III–V/silicon/Si_3_N_4_ laser presents high-temperature stability where the wavelength drift is only 10.46 pm/℃, over seven times smaller than that of typical InP/silicon heterogeneous lasers. Due to the ultra-low propagation loss of Si_3_N_4_ ranging from 0.1 dB/cm to 0.001 dB/cm, an extended long grating cavity can be realized, leading to Lorentzian linewidths as low as 400 Hz and output powers as high as 20 mW [61]. Due to the large transparency window of Si_3_N_4_, operation wavelength around 990 nm, well below the silicon bandgap, can be realized, which is promising to revolutionize many fields including displays, volumetric light projection, and AR/VR [62].

On the other side of the spectrum, the mid-infrared spectral range has become increasingly important due to its potential applications in spectroscopy, gas sensing, thermal imaging, and free-space communication. By adding a 400 nm-thick SiN_*x*_ layer between the 1.5 μm-thick top silicon and the 3 μm-thick BOX layer to avoid the strong optical absorption in SiO_2_ at the mid-infrared regime, a heterogeneous quantum cascade laser (QCL) was demonstrated with a wavelength of 4.8 μm and pulsed mode operation up to 100 °C (Fig. 3c) [63].

Furthermore, the wafer bonding technology allows for versatile management of the gain materials for the best achievable on-chip performance. A notable example is using QD active region for lower threshold current density, higher temperature stability, defect insensitivity, better immunity to fabrication defects, and reduced reflection sensitivity. Device design and process optimization have been actively conducted to replace InP-based quantum well (QW) epitaxial material with GaAs-based QD epitaxial material since 2016 [64]. In 2019, Zhang et al*.* presented a compact low-threshold microring laser with heterogeneously integrated QD epitaxial materials as the active layer, as shown in Fig. 3d. CW lasing was sustained up to 70 ºC [65]. In 2021, robust DFB lasers (Fig. 3e) have been demonstrated, with a 3-dB modulation bandwidth of 13 GHz, a threshold current of 4 mA, an SMSR of 60 dB, and a fundamental linewidth of 26 kHz [66]. While this field is still in an embryonic stage, the synergistic relationship between the III–V QDs and silicon through heterogeneous integration shows superior devices exceeding what is achievable with purely III–V QD devices. Lessons can also be leveraged through InP-based heterogeneous integration to both improve performance and reduce cost.

Furthermore, another important aspect of QD lasers is the small linewidth enhancement factor (α-factor), which is beneficial to a reduced reflection sensitivity. It has been experimentally demonstrated that QD lasers can exhibit over 100,000 × increase in the critical feedback level compared to that of QWs, such that coherence collapse does not occur even with 90% of the light reflected in the laser [67]. As a proof-of-concept demonstration, 25 Gb/s data-links isolator-free modulation with a metal–oxide–semiconductor capacitor microring modulator has been achieved [68]. This isolator-free laser source eliminates the need for co-packaged/integrated optical isolators while still maintaining the required feedback tolerance, significantly reducing the packaging complexities and enhancing the integration density for future PICs.

In addition to the versatile selection of bonded materials for gain, modulation, detection, etc., the heterogeneous integration also provides new degrees of freedom in designing novel device structures for broader and emerging applications. The ultra-low loss waveguides can form high-*Q* passive cavity architectures. Tunable, narrow-band filtering functionalities can thus be realized, pushing narrow linewidth device performance well beyond the abilities of their monolithic counterparts. As an example, a Lorentzian linewidth of 220 Hz has been achieved with a 110 nm wide wavelength tuning range using a coupled-triple-ring structure, as shown in Fig. 3f [69]. Even smaller Lorentzian linewidths (95 Hz) with a broader tuning range (120 nm) has been demonstrated with coupled-quadruple-ring structures [70]. Recently, Xiang et al*.* reported heterogeneously integrated laser soliton microcombs combining both DFB lasers and Si_3_N_4_ micro-resonators on a monolithic silicon substrate (Fig. 3g) [21]. A Lorentzian linewidth of 0.04 Hz has been realized through laser self-injection locking via a high-*Q* micro-resonator in single-soliton states [71]. Using on-chip electrical control of the microcomb laser relative optical phase, single-soliton microcombs with a free spectral range (FSR) of 100 GHz could be realized [21].

### Monolithic integration

Parallel to the success of heterogeneous integration, monolithic integration through direct epitaxial growth is another elegant path to integrating III–V gain materials onto the silicon substrates [72]. This technique provides natural heatsinking and is more economically favorable. However, the large mismatch of lattice constants, thermal expansion coefficients, and polarities between the silicon and the III–V materials lead to high densities of crystalline defects, including primarily threading dislocations (TDs), stacking faults (STs), misfit dislocations (MDs), and antiphase domains (APDs). These defects act as nonradiative recombination centers that grow with device operation, severely limiting the luminescence efficiency and the device lifetime. Central to solving these challenges is to reduce defects in the semiconductor material itself and improve defect tolerance of the active region [73].

To improve the defect tolerance of the active region, replacing the QW structure with QDs greatly suppresses lateral diffusion and non-radiative growth of defects. To reduce the number of TDs, post-growth thermal cycle annealing (TCA), insertion of dislocation filter layers (DFLs), and growth of compositionally graded buffers (*e.g.*, SiGe, GaP) to bridge the lattice constant between silicon and III–V have been heavily investigated. Notably, asymmetric step-graded (ASG) filters can efficiently reduce the TD density (TDD) to as low as 1 × 10^6^ cm^−2^ within a 2.55 µm GaAs virtual substrate grown on (001) silicon [74]. To reduce the MDs, thin strained QWs were inserted as trapping layers (TLs) above and below the active region, which efficiently eliminated 90% of MDs away from the active region [75]. To alleviate the effects of APDs, miscut silicon substrates with a 4°–6° offcut angle have traditionally been employed to form a prominent double-stepped silicon surface, but recent breakthroughs with on-axis (001) CMOS compatible silicon substrates have demonstrated comparable or better performance. To date, 300 mm GaP/silicon templates (NAsP_III/V_ GmbH) are commercially available with no APDs. In a parallel effort, nano-patterned V-grooved (111) surfaces can help achieve APD-free III–V coalescence [76]. In addition, selective-area hetero-epitaxy offers additional control over the strain relaxation process by restricting the epitaxial growth in pre-defined regions with appropriate substrate patterning. During the aspect ratio trapping (ART) process, dielectric stripes with a sufficiently large aspect ratio can provide complete defect necking and essentially trap all defects within the bottom areas. In conjunction with epitaxial lateral overgrowth (ELOG) [77, 78], large-area APD-free III–V materials can be epitaxially grown on silicon. These growth innovations and corresponding device milestones can be summarized into five development stages, as detailed in the following part.

**Fig. 4:**
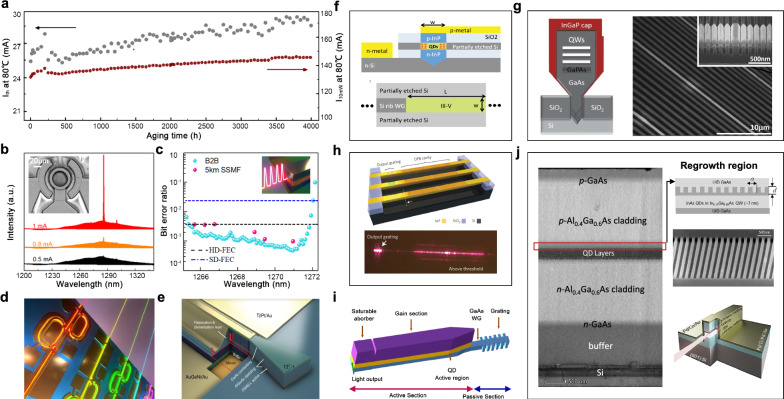
**Monolithic integration**. **a** Robust laser operation at 80 °C with >1 M hour extrapolated lifetime. Adapted with permission from [84], copyright 2021, OPG. Individual QD lasers directly grown on silicon with **b** submilliamp threshold microring lasers, **c** mode-locked lasers, **d** coupled cavity tunable lasers and e DFB lasers. Adapted with permission from [85–88], copyright 2017 & 2019, OPG. **f** Schematic illustration of a selectively grown III–V cavity onto the handle wafer of an SOI substrate. Selective growth of box-shaped III-V materials on silicon for **g** a GaAs based nano-ridge laser array and h InP based DFB laser array. Adapted with permission from [89–91], copyright 2015 & 2017, OPG & NPG. **i** A monolithic offset QD integration platform where the light from QD active region is coupled to passive GaAs waveguides. Adapted with permission from [93], copyright 2020, OPG. **j** A regrown III-V QD-DFB laser that can combine different bandgaps into one optical chip. Adapted with permission from [94], copyright 2020, WILEY

In Gen-I, pioneering work in UCL achieved the first 1.3 μm CW lasing at room temperature using miscut silicon substrates and Ge buffer [79]. In Gen-II, various efforts have been explored to achieve on-axis (001) CMOS compatible silicon substrates, including GaAs buffer on APD-free GaP/Si(001) [80], nano-patterned V-grooved (111) surfaces [76], (111)-faceted silicon hollow structures [81], a sophisticated silicon surface preparation [82], etc., to grow APD-free III–V buffers on silicon. In Gen-III, extrapolated lifetime (defined as the time required to double the initial threshold current) has been improved to 1 M h at room temperature, and 5000 h at 60 °C by reducing the TDD in the GaAs buffer layers to 7 × 10^6^ cm^−2^ [83]. In Gen-IV, the *p*-type modulation doping (pMD) process was employed to compensate for the carrier escape at high temperature, giving rise to a higher material gain, a lower slope efficiency, a higher threshold current and a 70,000-h extrapolated lifetime at 60° [83]. In Gen-V, the synergistic effects of TLs [75] and ASG filters [74] greatly improve the high temperature. Minimum degradation after more than 4000 h of constant current stress was demonstrated, with an extrapolated lifetime of over 22 years. This marks an important milestone for commercial use in data centers (Fig. 4a) [84].

To date, the performance of individual devices for QD lasers directly grown on silicon substrates has been significantly improved. To name a few accomplishments, submilliamp threshold QD microring laser with CW lasing up to 100 °C was demonstrated (Fig. 4b) [85], QD mode-locked lasers with a wide mode-locking regime and timing jitter of 82.7 fs with an aggregate total transmission capacity of 4.1 Tb/s (Fig. 4c) was demonstrated [86], QD coupled cavity tunable lasers with a simple, integrable architecture to achieve over 45 dB SMSR without involving regrowth steps or subwavelength grating lithography (Fig. 4d) was demonstrated [87], and QD DFB lasers with stable single mode operation and an SMSR as high as 50 dB (Fig. 4e), was demonstrated [88]. Several paths have also been explored for active–passive coupling and co-integration of QD lasers to the other parts of silicon photonics. For a pure monolithic approach, light generated in the QD section can be coupled to either the silicon waveguide in an SOI substrate or within the III–V layers.

*For light coupling with other silicon photonic components*, growing the III–V gain materials in pockets in a butt-coupled configuration is actively pursued by several groups and could provide the best economies of scale. Back in 2015, Liu et al. proposed that the III–V cavity could be selectively grown onto the handle wafer of an SOI substrate, and the height of the active region can be aligned with the silicon waveguide layer to maximize optical coupling, as shown in Fig. 4f [89]. Alternatively, in a buffer-less selective growth of III–V on silicon, the shape of the material growing out of the trench can be engineered with a box shape, where the lateral width of the box is on the order of a micrometer, comparable to the typical width of a “classical” III–V laser. Experimentally, optically pumped nano-ridge lasers on patterned 300 mm silicon have been demonstrated with GaAs based materials (Fig. 4g) [90], and optically pumped DFB laser arrays have been demonstrated with InP-based materials (Fig. 4h) [91]. However, electrical injection of such structures is challenging. Since current injection would go through the highly defective III–V/silicon interface, a large resistance was a concern. Nevertheless, recently high performance epitaxially grown QD lasers grown on 300 mm substrates with low resistance were demonstrated [92]. Monolithic integration with butt-coupled silicon waveguides has also been achieved with a maximum output power of 6.8 mW and estimated coupling efficiency of approximately -7.35 dB [44].

*For light coupling with III–V waveguide layers*, all the photonic functionalities are performed in the III–V materials, and the silicon wafer is only used as a handle substrate. Similar to the case of photonic integration on InP, this approach can be achieved by regrowth, offset QDs, intermixing, etc. Following this direction, a monolithic offset QD integration platform has been built to couple light from QD active region to passive GaAs waveguides (Fig. 4i) [93]. High-quality MBE regrowth capability in QD-based materials has been demonstrated, which promises to combine different bandgaps into one optical chip (Fig. 4j) [94]. However, the relatively small index contrast provided by III–V waveguides compared to that of the SOI material system reduces optical confinement and dense integration prospects to some extent.

In a nutshell, with lifetimes entering the realm of commercial relevance, monolithic integration promises to scale photonic integrated circuits to 300 mm or even 450 mm diameter wafer size for high volume applications. However, fully integrated photonic circuits and device integration with other advanced silicon-based photonic components are still in the initial stages. More work needs to be done for active–passive coupling and co-integration in future research.

## Applications of on-chip lasers

Silicon photonics has become a prominent platform requiring numerous integrated components with myriad interconnects and long routing paths. Considering the overall loss budget, it is paramount to minimize the losses and maximize the gain as much as possible.

In terms of waveguide loss, typical silicon waveguide loss on the order of 0.1 dB/cm to 1 dB/cm in datacom applications can hardly meet the requirement of low-loss waveguide circuits in quantum photonic applications, such as high-key-rate QKD, high-rate Boson sampling, efficient and generation of cluster states [95]. To that end, various approaches have been taken, including photoresist reflow and optimized etch chemistry, reducing etch depth and increasing waveguide width, using ultra-low loss silicon nitride (Si_3_N_4_) waveguides, high-resolution immersion lithography, and H_2_ thermal annealing. Recent progress has demonstrated waveguide losses down to < 0.1 dB/cm in silicon and < 0.1 dB/m for Si_3_N_4_ [96, 97].

In terms of the integration configuration, 2.5/3D integration can be used to reduce the length of the electrical bonded wires and the attenuation of electrical signals and associated losses. In terms of the active regions, direct bandgap, high gain, and strong optical and carrier confinement material is required for low lasing threshold and high wall-plug efficiency.

In terms of losses within the integrated lasers, they can be either associated with the resonant cavities, scattering, spontaneous emission, photon parasitic absorption, or non-radiative recombination phenomena. Resonant cavity losses are attributed to the limited reflectivity of the cavity mirrors, and it along with scattering losses and parasite absorption dictate the modal photon lifetime within the cavity. Non-radiative recombination in lasers takes place predominantly as Auger recombination. This multicarrier nonradiative recombination process is highly thermally activated and represents the major roadblock to improving the efficiency of semiconductor lasers. Scattering, mainly Raman scattering, is another major source of losses in both integrated semiconductor lasers and silicon waveguides. This effect is attributed to crystal defects and side-wall roughness that increases with the depth of the etched rib SOI waveguides.

In terms of the coupling losses between the on-chip III–V lasers and the silicon circuits. In hybrid integration, minimizing the optical losses is achieved by butt-coupling lasers directly to silicon PICs, or using fiber to bridge the two chips together. In heterogenous integration, the lithography-defined tapers can shape the hybrid active mode to couple into a passive Si waveguide mode through evanescent coupling. The elimination of chip-to-chip coupling loss and availability of on-chip gain from heterogeneous integration can achieve high overall efficiency with coupling losses lower than 0.5 dB/interface [43].

In terms of the mismatch losses and insertion losses when interfacing different components in the circuitry, introducing on-chip lasers can remove the fiber-to-chip optical coupler between the laser and silicon PICs, leading to less overall loss and improved link budget. In datacom, modulators are associated with a minimum insertion loss of 3 dB (6 dB in practice) on top of a 1-to-2 dB coupling loss associated with an off-chip coupled laser [98]. Having compact, densely integrated on-chip lasers that could be directly modulated would likely save at least 6 dB in power consumption over the most advanced datacom links today.

With that being said, dense device integration with on-chip lasers will be critical for a number of envisioned future applications. In what follows, we will discuss the benefits and potential implementation of on-chip lasers in various functionalized PICs, including optical communications and interconnects, optical phased array-based LiDAR, sensors for chemical and biological analysis, integrated quantum technologies, and optical computing.

### Optical communication and interconnects

Currently, the biggest driver in further development of silicon photonics is still datacom [99–102]. A pioneering explorer is Intel. Having the heterogeneously integrated silicon lasers in its portfolio, Intel has shipped 100 Gb/s CWDM4 (Coarse Wavelength Division Multiplexing 4) transceivers over one million units annually, and several new products are ramping, such as 200G FR4 and 400G DR4 pluggable transceivers [100, 101]. In 2022, optical transceivers with integrated lasers with a capacity of 800G are demonstrated, which comprised of an 8-channel transmitter with 100Gbps Mach–Zehnder Interferometer modulators (MZM) and on-chip high-power DFB lasers [103].

### Optical phased array (OPA)-based LiDAR

Dense device integration with on-chip lasers is critical for several other envisioned future applications. LiDAR is one obvious example: to popularize the chip with dozens of coherent receivers and dozens of semiconductor optical amplifiers that boost the output power into free space, dense integration of different functionalities is a prerequisite [104]. In recent years, integrated optical phased arrays (OPA)-based frequency modulated continuous wave (FMCW) LiDARs have attracted intensive attention due to their potential to realize long detection range, directly velocity measurement, robust interference immunity, low power consumption, and cost-effective LiDAR systems on a tiny chip [105, 106].

**Fig. 5:**
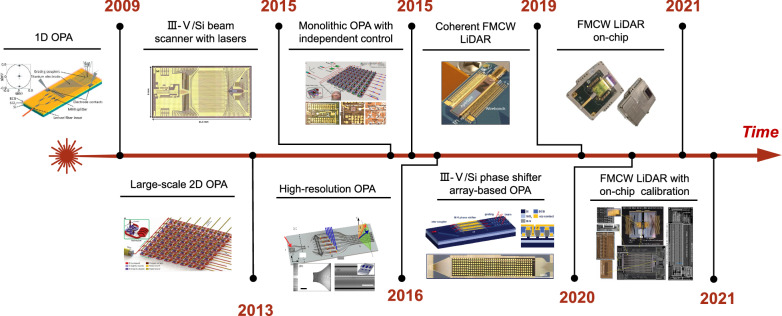
**Development of silicon photonics-based LiDAR**. The first silicon photonics-based OPA. Adapted with permission from [108], copyright 2009, OPG. A large-scale two-dimensional OPA with complex far-field patterns. Adapted with permission from [109], copyright 2013, NPG. III-V/ silicon beam scanner with lasers. Adapted with permission from [111], copyright 2015, OPG. Monolithic 2D OPA with independent integrated electric control. Adapted with permission from [112], copyright 2015, OPG. High-resolution OPA based on non-uniform waveguide arrays. Adapted with permission from [113], copyright 2016, OPG. Extremely low static power consumption OPA system based on III-V/silicon phase shifter arrays. Adapted with permission from [114], copyright 2019, OPG. The first packaged coherent OPA-based LiDAR on a silicon photonic platform. Adapted with permission from [115], copyright 2019, IEEE. Mobile Eye’s integrated FMCW LiDAR. Image credit: Intel [116]. The first large-scale OPA-based silicon photonic LiDAR with on-chip array calibration capability. Adapted with permission from [117], copyright 2021, IEEE

The progression of OPA-based FMCW LiDAR is presented in Fig. 5. As a core component of LiDAR, OPA consists of an array of coherent antennas where amplitudes and phases can be controlled to generate arbitrarily shaped emitted radiation patterns through interference [107]. The phased array technology has been successfully demonstrated in radio waves more than a contrary ago, but it was brought to the optical frequency until the early 90 s. In 2009, the first silicon photonics-based OPA was demonstrated by Ghent University [108]. A lateral steering range of 2.3° was achieved at 1550 nm by adjusting the heating electrodes and a longitudinal steering range of 14.1° was obtained by tuning the wavelength from 1500 to 1600 nm. In 2013, J. Sun et al. at MIT demonstrated a large-scale two-dimensional (2D) OPA with complex far-field patterns [109], where a tunable heater was added to achieve a 12° × 12° field of view [110]. In 2015, Hulme et al*.* at UCSB presented the first fully integrated 2D free-space beam-steering chip on the heterogeneous platform. The PIC incorporates a total of 164 components with 9 different component types, including 2 tunable lasers, 34 amplifiers, and 32 photodiodes [111]. In the same year, Abediasl et al. at USC demonstrated the first monolithic 2D OPA driven by CMOS electronics in a commercial CMOS fabrication process [112]. The compact chip contains over 300 optical components and over 74,000 electrical components. Each optical component has an independent amplitude and phase controller to generate arbitrary radiative direction patterns. Nevertheless, these reported OPA systems are mainly based on uniform waveguides or grating arrays with strong constructive interference when the uniform emitter spacing is greater than about half a wavelength, causing a limitation on resolution and beam steering range. In 2016, in order to achieve an ultra-wide beam steering range and high resolution, Intel proposed and demonstrated a non-uniform sparse waveguide array-based OPA [113]. The OPA exhibits excellent performance with a lateral steering range of 80°, a longitudinal steering range of 17°, and a record small divergence of 0.14° in small spot size. Later, intel’s LiDAR-on-a-chip design was announced for autonomous vehicles, with more than 6,000 individual components integrated into these LiDAR PICs. In 2019, Xie et al*.* demonstrated an OPA system based on III–V/silicon phase shifter arrays with a pitch of under 4 μm, achieving 2D steerable far-field beams with a beam width of 0.78° × 0.02° and a field of view of 22° × 28°. Based on the heterogeneous silicon photonics platform, this OPA for chip-scale LiDAR system has extremely low static power consumption (< 3 nW), and high-speed operation (> 1 GHz) [114]. In the same year, Watt’s group at MIT reported the first electrically and optically packaged coherent OPA-based LiDAR on a silicon photonics platform [115]. The one-dimensional 512-elements OPA was demonstrated with record low power operation (< 1mW total) and high-speed beam steering (< 30 μs phase shifter time constant). The packaged coherent OPA-based LiDAR system has a 2D detection range of 185 m and a 3D scanning range of 10 m on a measured diffuse target. At CES 2021, Mobile Eye launched an integrated FMCW LiDAR based on silicon photonics platform, enabling 184 vertical lines per scan on a tiny chip [116]. This prototype integrates the active and passive components and driver circuits required for LiDAR, making silicon photonics-based LiDAR a big step forward in commercialization. Thereafter, the first large-scale OPA-based silicon photonic LiDAR with on-chip array calibration capability was proposed. Array calibration was incorporated to overcome the inherent mismatches across the optical phased array, which further improved the reliability of silicon photonics-based LiDAR [117].

While silicon photonics-based FMCW LiDAR systems have made a variety of breakthroughs, several challenges remain before their wide adoption in automotive driving. *For on-chip light sources*, there are stringent requirements on narrow bandwidth, high-frequency modulation linearity, sufficient speed of the tunning response, and low cost for high-volume production. Since redundancy is needed to mitigate risks in case of fallback and to guarantee coverage of all adverse conditions, the detection range of the LiDAR deployed in a real system should exceed 200 m. Thus, the emitted power of the antennas is desired to support the maximum permissible radiation power set by the laser safety regulation (∼10 dBm for IEC60825-1 Class 1 in C-band [118]), which means the emitted power of the laser should support sufficient output power over 10 dBm plus the power loss in light paths. *For losses within OPAs*, the loss of phase-shifters limits the range of current OPA-based LiDAR to sub-1 km applications. In this regard, Si_3_N_4_ can be deployed to achieve OPA with low optical propagation loss, temperature insensitivity, and high optical power-handling capability by heterogeneous integration technology [119, 120]. In addition, dense integration of on-chip semiconductor optical amplifiers (SOAs) placed after the phase-shifters is required such that the OPA losses can be efficiently compensated [121]. The density requirements on OPAs are at least an order of magnitude higher than in typical telecom/datacom products, typically approaching a half-wavelength pitch. Therefore, the amplifier integration must be exceedingly dense to address this application. Furthermore, for the implementation of LiDAR in commercial vehicles, at least an order of magnitude cost reduction is required compared to the current solutions. The only possible way is dense photonic integration with on-chip lasers and amplifiers, and volume manufacturing at 300 mm semiconductor foundries with wafer-scale testing.

To date, OPA-based FMCW LiDARs with a 200 m detection range, high immunity to interference, centimeter-level size, and low cost on silicon photonics platform are still under investigation. Through dense device integration with on-chip gains, it is feasible to build an advanced LiDAR system on a tiny chip for automotive driving in the near future.

### Integrated photonic sensors for chemical and biological analysis

The Covid-19 pandemic has spawned intense interest to explore ultra-high accurate and portable biochemical sensors [122], of which silicon-based photonic sensors stand out as one of the most promising candidates [123, 124]. The photonic sensors use optical waveguides as a sensing element. Light evanescently coupled into the upper and side cladding can interact with the analyte, and change the optical or electrical properties accordingly [125]. Silicon photonic waveguide-based sensors can be classified into spectroscopic sensing and refractive-index-based sensing [126]. The former mainly includes absorption spectroscopy and Raman spectroscopy, while the latter mainly includes microring resonator (MRR) based sensors, interferometric-based sensors [127], and photonic crystal-based sensors [128].

#### Spectroscopic sensing

**Fig. 6:**
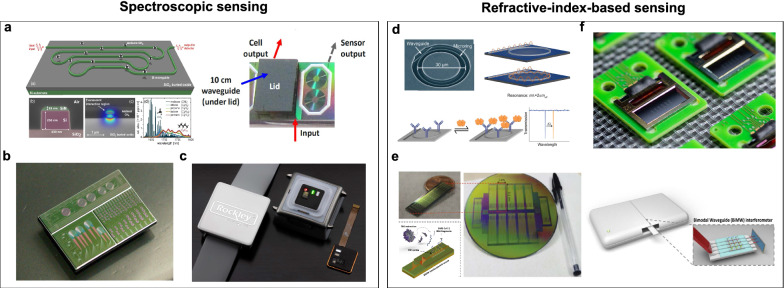
**Silicon photonic sensors for chemical and biological analysis**. Spectroscopic sensing, **a** The fully integrated packaged silicon photonic CH4 sensor. Adapted with permission from [129], copyright 2017, OPG. **b** The Chip-scale Sarin gas silicon photonics sensor. Adapted with permission from [126], copyright 2019, SPIE. **c** Wearable silicon photonics biomarker monitor based on Raman spectroscopy. Image credit: Rockley [130]. Refractive-index-based sensing. **d** Genalyte’s biosensor based on microring arrays. Adapted with permission from [131], copyright 2015, ACS. **e** A nanophotonic biosensor based on the BiMW interferometer. Adapted with permission from [132], copyright 2021, IOP. **f** An integrated photonic biosensor prototype for cancer diagnosis Image credit: PHIX [133]

For spectroscopic sensing, the first fully integrated packaged silicon photonic trace gas sensor (Fig. 6a) was demonstrated by Tombez et al*.* at IBM in 2017 [129]. A 10 cm-long transverse magneticwave-operated waveguide with an evanescent field ratio of 26.3% was employed to enhance the absorption spectroscopy of methane (CH_4_). With a λ = 1651 nm III–V-based laser integrated on the silicon chip through the flip-chip bonding, the sensor achieves a high sensitivity of 5 ppmv, low power consumption of 0.6 mW, and a low cost of 0.25 k USD. Nevertheless, this sensor relies on external PCB (printed circuit board) based driver circuits which degrade its integrity and compactness. In 2018, AIM Photonics developed a chip-scale Sarin gas silicon photonic sensor (Fig. 6b) [126]. The sensing sensitivity was enhanced by adopting a long-path on-chip waveguide and coating it with a polymer to preconcentrate the gas sample. In 2021, Rockley photonics demonstrated a non-invasive, portable, and wearable silicon photonics biomarker monitor based on Raman spectroscopy (Fig. 6c), which can detect a broad range of physiological measurements and health and wellness parameters, including core body temperature, body hydration, lactate, ethanol, urea, glucose, etc. [130]. By replacing the traditional LED (light-emitting diode) technology with an array of lasers and integrated spectrometers covering the entire infrared and visible light spectra, the sensing chip has superior advantages in terms of range, accuracy, efficiency, and has been widely adopted in wearable and mobile products.

#### Refractive-index-based sensing

For the refractive-index-based sensing with silicon photonic, a low limit of detection (LOD), can be achieved down to 10^–8^ refractive index unit (RIU) since the sensors are usually performed at a single wavelength. The main challenge is to modify the surface of the sensing chip at the micro- and nano-size so that it can capture and monitor specific molecules in a complex environment. In *Genalyte* (a U.S. sensing startup), surface biofunctionalization of different microrings can be utilized to capture different proteins or DNA for simultaneous detection (Fig. 6d) [131]. Following this method, the Covid-19 detection system with 30 min diagnosis was commercialized, which significantly reduced the detection time during the pandemic. However, the off-chip light sources and spectrometers lead to a bulky footprint and a high cost. In 2020, Ruiz-Vega et al*.* proposed a nanophotonic biosensor for point-of-care (POC) Covid-19 diagnostics and coronavirus surveillance (Fig. 6e) [132]. Based on the bimodal waveguide (BiMW) interferometer, minute concentrations (aM-fM levels) of RNA can be detected within 30 min, with an accuracy comparable to the standard polymerase chain reaction (PCR)-based assays. Following this method, PHIX demonstrated a photonic biosensor prototype (Fig. 6f) that integrates Si_3_N_4_ photonic integrated circuits with on-chip VCSELs (vertical-cavity surface-emitting lasers), PDs, electronic PCBs, and microfluidic interfaces [133]. The wafer-level manufacturing capability can significantly lower the price and reduce the footprint through mass production, making the POC device a promising candidate for cancer diagnosis and treatment monitoring.

Over the last few decades, silicon-based photonic biochemical sensing platforms have made significant advances in chip-scale integration and miniaturization for portable, label-free bio-diagnosis and chemical analysis. Looking forward, with an integrated light source, along with the continued advancement of on-chip spectroscopy, microfluidics, surface-chemistry, and packaging technologies, silicon-photonics biochemical sensors will truly realize “lab-on-chip” and provide greater insights into disease progression and management in the near future.

### Integrated quantum technologies on photonic chips

Quantum photonic technologies leverage quantum superposition and entanglement of light to realize extreme superiority in communication, information processing, computing, simulation, and sensing applications [134, 135]. For example, quantum computer *Jiuzhang* achieved processing of Boson sampling 10^14^-fold faster than supercomputers [136] while Yin et al. demonstrated the Micius satellite-based entanglement distribution to receiver stations separated by more than 1200 km, which is important for secure communications [137]. These benefits of quantum technologies have been extensively explored theoretically and experimentally, with significant progress in several platforms, including superconductors, ion traps, neutral atoms, and silicon photonics [135].

**Fig. 7:**
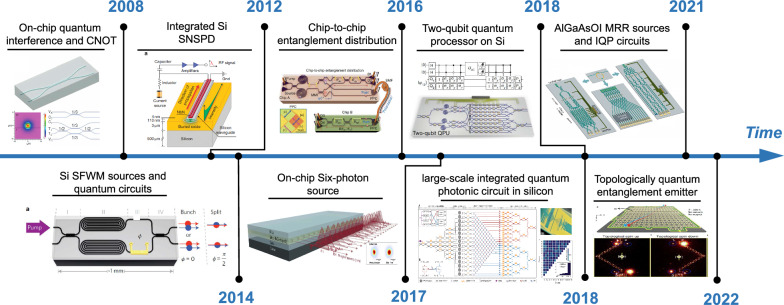
**Key milestones in silicon-based IQP. On-chip quantum interference and integrated CNOT gate on SiO2**. Adapted with permission from [138], copyright 2008, AAAS. Integrated silicon SNSPD. Adapted with permission from [139], copyright 2012, NPG. The first integration of single-photon sources with quantum circuits on silicon. Adapted with permission from [140], copyright 2014, NPG. Chip-to-chip entanglement distribution system between two silicon chips. Adapted with permission from [141], copyright 2016, OPG. On-chip generation of six photons. Adapted with permission from [142], copyright 2017, OPG. The fully programmable two-qubit quantum processor on silicon [143], large-scale silicon IQP device comprising over 670 optical components. Adapted with permission from [18], copyright 2018, AAAS. The tunable AlGaAs-on-insulator (AlGaAsOI) MRR entangled-photon-pair source and all-on-chip quantum photonic circuits. Adapted with permission from [144], copyright 2021, APS. Topologically quantum entanglement emitter with a high tolerance of fabrication. Adapted with permission from [145], copyright 2022, NPG

As is shown in the key milestones in Fig. 7, the first integrated quantum photonic (IQP) technologies comprised a two-photon interference and a controlled-NOT (CNOT) entangling gate in the silica-on-insulator platform in 2008 [138]. This breakthrough lays the foundation for the development of IQPs with stability, controllability, and compact size. However, due to silica's low refractive index, silica-based devices and waveguide bending radius are relatively large, making them impractical for large-scale integration. More focus has shifted to silicon-based IQP thereafter, including single-photon sources (SPSs), quantum state encoding and manipulation, single-photon detectors (SPDs), and quantum communication, information processing, and simulation.

#### Integrated silicon-based SPSs

For an SPS, photon pairs can be generated through the nonlinear optical process, such as spontaneous four-wave mixing (SFWM) and spontaneous parametric down-conversion (SPDC). The simplest silicon-based photon-pair source relies on SFWM by pumping a well-designed centimeter-length silicon waveguide. This type of photon-pair source can be easily integrated into large arrays and exhibits a high purity, heralding efficiency and indistinguishability. In 2020, a spiraled multi-mode (MM) waveguide-based photon-pair source was demonstrated with a spectral purity of 0.9904 ± 0.0006, mutual indistinguishability of 0.987 ± 0.002, and > 90% intrinsic heralding efficiency [146]. However, the produced photon pairs by waveguide pumping are highly correlated in frequency. Narrowband spectrum filtering is required to improve their spectral purity, resulting in a large reduction in photon rates and heralding efficiency.

Alternatively, photon-pair sources can be generated based on MRRs. In 2020, multiple high-quality single photons in silicon have been generated through an MRR-based source array, which shows a purity of 90%, an indistinguishability of 91%, and a heralding efficiency of 50% [147]. However, non-uniformity and fabrication imperfections remain an issue. Later, Dai et al. at Peking University realized a topologically protected integrated quantum entanglement light source based on a total of 280 silicon-based microrings [145]. When the photons are transmitted along the topological boundary, loss-free transmission can be maintained even when certain defects and imperfect structures are present. The experimental results show 96.8% high fidelity and 96.2% high purity from the topological quantum-protected Einstein–Podolsky–Rosen (EPR) entangled state. This makes it possible for quantum photonic chips to operate accurately and efficiently despite fabrication imperfections.

#### Quantum state encoding and manipulation on silicon-based photonic chips

On-chip quantum state encoding and manipulation are at the heart of quantum information processing. The single photon of light as quantum information carriers can be encoded and manipulated in various degrees of free (DoFs), including position/path, polarization, frequency, and spatial and temporal modes. All these manipulations can be implemented on a silicon-based photonic chip. Take path-encoding as an example, photons simultaneously locating over dual/multiple waveguides |k〉 form path-encoded qubit/qudit states, which can be arbitrarily generated, controlled, and measured using integrated reconfigurable Mach-Zehnder interferometer (MZI) [18, 140]. Other DoFs can be manipulated by Polarization Beam Splitter (PBS) [148], resonators [149], multimode waveguides, etc. [150]. A series of experiments have demonstrated that the generation, manipulation, and measurement of photons entanglement on a silicon platform is remarkably stable and efficient. In 2014, Silverstone et al. at the University of Bristol first demonstrated reconfigurable waveguide circuits with on-chip identical photon sources in SOI [140]. The IQP chip combines two four-wave mixing sources in an interferometer with a thermo-optically phase shifter. The device shows the ability to generate and manipulate two-color (non-degenerate) or same-color (degenerate) path-entangled or path-unentangled photon pairs. 100.0 ± 0.4% visibility quantum interference on-chip and 95 ± 4% off-chip can be observed. In 2018, a state-of-the-art large-scale silicon IQP device with more than 670 optical components was demonstrated for the generation, manipulation, and measurement of multidimensional entanglement [18]. 16 identical SFWM single-photon sources were integrated on the chip and 15 × 15 entangled states were demonstrated. These large-scale IQP devices show that very large-scale integration of quantum photonic circuits with millions of components on silicon is conceivable.

#### Integrated SPDs

On-chip SPDs are the core device to read out quantum information of photons. Avalanche photodiodes, superconducting nanowire single-photon detectors (SNSPDs), and transition edge sensors (TESs) are available for single-photon detection. SNSPD is considered as a promising candidate due to its superior performances in detection efficiency, time jitter and dark noise [151]. In 2012, Pernice et al*.* demonstrated a traveling wave integrated SNSPD that operates at 1550 nm wavelength with high detection efficiency up to 91% and a low-timing jitter of 18 ps. The SNSPD is embedded in scalable silicon photonic circuits and is valuable for high-fidelity manipulation and evaluation of quantum states of light on a chip. However, SNSPDs only work at cryogenic temperatures and on-chip SPD is still an ongoing challenge.

#### Quantum communication, information processing, and simulation

Quantum mechanics enable a variety of novelty applications, including perfectly secure quantum communication, efficient quantum information process, and simulation. The future-envisioned global-scale quantum communication network is connected by optical fibers and space links, where silicon IQP chips are expected to reduce the physical footprint and power consumption, improve stability, and enable high-volume production.

**Fig. 8:**
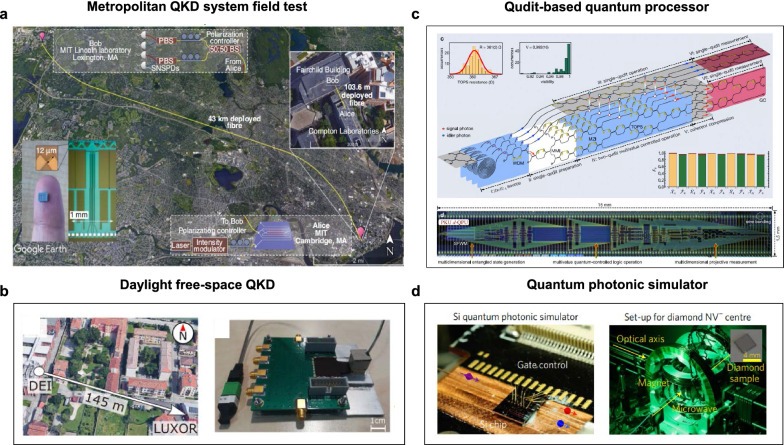
**Silicon-based integrated quantum photonic applications**. **a** The first field tests for a metropolitan QKD connected by optical fiber.Adapted with permission from [152]. copyright 2018, APS. **b** Daylight free-space QKD using silicon photonic circuits. Adapted with permission from [153], copyright 2021, NPG. **c** A programmable qudit-based quantum processor in silicon-based IQP circuits. Adapted with permission from [154], copyright 2022, NPG. **d** A quantum photonic simulator and diamond nitrogen-vacancy center. Adapted with permission from [155] copyright 2017, NPG

Using a high-speed silicon photonics-based encoder, Bunandar et al*.* at MIT demonstrated the first field tests for polarization-encoded QKD [152]. Figure 8a presents the aerial view of the intercity QKD field test. In the intercity test between the cities of Cambridge and Lexington, secret keys were generated at a rate of 157 kbps and a bit error rate of 2.8% was observed, connected by a 43 km dark fiber. The systems achieve composable secret key rates of 1.039 Mbps in a local test (on a 103.6-m fiber with a total emulated loss of 9.2 dB) and 157 kbps in an intercity metropolitan test (on a 43-km fiber with 16.4 dB loss).

For space links, Avesani et al. presented a silicon photonic prototype for daylight QKD operating at 1550 nm and its field test, as shown in Fig. 8b [153]. The QKD source and detection units are linked through a free-space channel built between the Department of Information Engineering (DEI) and the Luxor Laboratory (LUXOR). The experiment results show a quantum bit error rate of around 0.5% and an averaged secret key rate of 30 kbps during a whole sunny day, which is promising to design quantum optical payloads for future satellite missions.

For a programmable qudit-based quantum processor, by reprogramming the integrated reconfigurable MZIs on a silicon photonics platform, basic Fourier transform algorithms can be implemented in quaternary [154]. As shown in Fig. 8c, qudit-based quantum computation is proven to enhance quantum parallelism in terms of computational capacity, accuracy, and efficiency over its qubit-based quantum computing counterparts.

Furthermore, Quantum simulation is believed to be one of the most promising applications of quantum computers as it can efficiently calculate Hamiltonian spectra, a problem often intractable on classical machines. Using a silicon-photonics quantum simulator to learn the Hamiltonian of the diamond nitrogen-vacancy center (Fig. 8d) [155], the reprogrammable quantum chip demonstrated the quantum Hamiltonian learning (QHL) capability of validating Hamiltonian models and verifying quantum devices.

Thanks to the advanced CMOS fabrication process, silicon-based photonic quantum chips have made great progress in the last decade. It is more realistic to realize IQP chips by integrating optical components made with various material systems [156]. For example, QD SPSs based on III–V materials offer the potential for deterministic generation of single photons [157] and SNSPDs fabricated on superconducting materials can be enhanced by active feedback operations of on-chip photonic architectures [156]. However, a fully integrated chip-based quantum chip has not yet been realized. Integrating silicon wafers with light sources & photodetectors made of other semiconductors remains a challenge. The related research is still in the fundamental stages. However, if light sources are already being integrated into silicon photonics for classical applications (mainly in datacom) with established processes (there are both heterogeneous, and monolithic approaches), then it makes sense to grant them extra consideration in designing future quantum computation platforms for practical deployment and commercialization.

### Optical computing

Efficient hardware, dominated by electrical computing, provides the necessary ingredients for massive computation in the era of artificial intelligence (AI). But as devices continue to shrink in size, transistor leakage turns into a great concern, which poses an upper limit to performance [158]. As a complementary solution to electrical computing, optical computing provides high-speed computations with ultra-low power consumption and massive parallelism [22, 159]. This can effectively accelerate mathematical operations, including matrix–vector multiplication (MVM), which is a fundamental operation in artificial neural networks [160]. The methods for implementing MVM with optical devices, also known as optical MVM, fall into three main categories: the plane light conversion (PLC) method [161, 162], MZI method [22, 163], and wavelength division multiplexing (WDM) method [164, 165]. High integration density is of significance for high-performance hardware platforms. However, PLC approaches require considerable diffraction space to support large-scale inputs [166]. MZI approaches, which tend to demand a large number of MZI modules, can also occupy a considerable area [167]. WDM-MVM based on MRR arrays, on the other hand, offers distinct advantages for ultra-dense computing in optics. By driving a large number of WDM links with a small amount of energy using on-chip lasers, large-scale applications can be expected with mass production and low power consumption [168, 169].

**Fig. 9:**
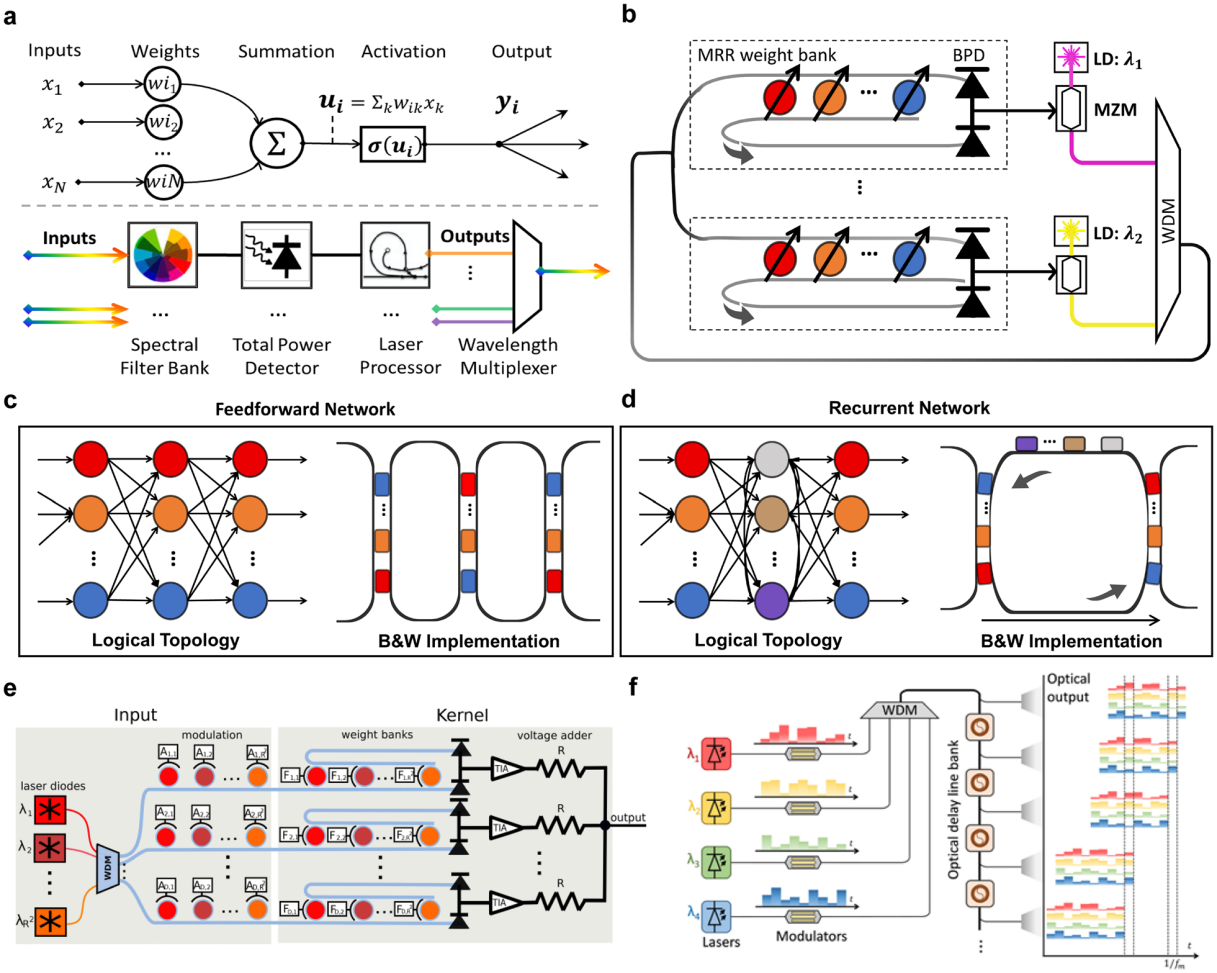
**Schematics of the B&W architecture for neural networks**. **a** The B&W architecture (bottom) shows parallels with the neuron model (top). Adapted with permission from [164], copyright 2014, OPG. **b** Concept of a B&W network with MRR arrays as the spectral filters. Adapted with permission from [165], copyright 2017, NPG. **c**–**d** Different mapping topologies for photonic neural networks, e.g., **c** feedforward network and **d** recurrent network. Adapted with permission from [174], copyright 2018, IEEE. **e** An optoelectrical architecture that performs a single convolution window. Adapted with permission from [175], copyright 2020, IEEE. **f** Conceptual layout of an optical patching scheme. Adapted with permission from [, copyright 2020, OPG^176^

In 2014, Tait et al*.* pioneered a feasible and representative architecture to achieve scalable, cascadable, and reliable photonic neural networks. This is known as the “broadcast-and-weight” (B&W) and has also been recognized as the cornerstone for WDM-MVM realization [164]. Later in 2017, Tait et al*.* correspondingly contrived a recurrent neural network on a silicon-photonic prototype, where each neuron, *i.e.*, the core component, is composed of an MRR array, a pair of balanced photodetectors, and a laser source, as illustrated in Fig. 9a, b [165]. In this manner, the input signals with various wavelengths can be weighted by the MRR array and then summed by the photodetector pairs to complete the MVM operation. Several well-developed technologies can help sustain reliability within a simple structure. In particular, the weight matrices of the photonic neural networks can be directly mapped onto MRR arrays [170], loaded and get fine-tuned at high speed (~ 54 GHz [171]) through standard electrical control [172, 173]. With this architecture, scaling up neural networks does not lead to a dramatic escalation in overhead since one single-mode fiber can accommodate multi-channel inputs. As proposed in 2017, it has the potential to yield a 294-fold speedup over the existing benchmarks [165]. It is worth noting that the laser source introduced to build distinct-wavelength interconnects between neurons and to counterbalance transmission losses between network layers is an indispensable component, which ensures scalability and cascadability.

**Fig. 10:**
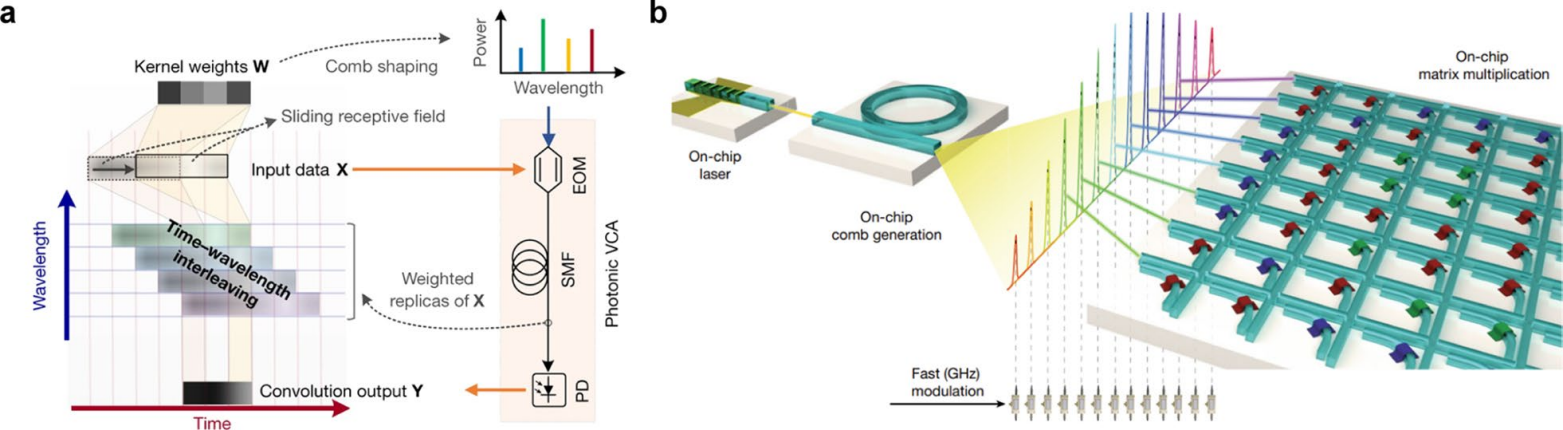
**a** Illustration of the photonic vector convolutional accelerator that interleaves temporal, wavelength and spatial dimensions. Adapted with permission from [178], copyright 2021, NPG. **b** A fully integrated photonic tensor core for parallel convolutional processing, taking advantage of the in-memory computing technology. Adapted with permission from [179], copyright 2021, NPG

Due to the competitive performance and delicate design, “B&W” and its variants have been extended to a variety of neural network topologies, including feedforward neural networks (FNN) and convolutional neural networks (CNN) [174–179]. FNNs and CNNs are research hotspots in the field of algorithms and have been widely used in many scenarios such as adaptive or massively parallel problems, but rely heavily on hardware capabilities [180]. To implement photonic FNNs, Peng et al*.* have suggested several mapping topologies on silicon photonics in 2018 as illustrated in Fig. 9c, d [174], providing a meaningful solution for achieving sophisticated AI models. Given that former architecture and mapping topologies, in 2019, Bangari et al. proposed a feasible optoelectrical architecture for CNN acceleration that was verified through simulations [175]. As shown in Fig. 9e, the scheme was designed for accelerating the convolution operations via the WDM-MVM method. An appreciable speedup ratio was ensured in way of combining the superiorities of digital electronics and analog photonics. However, it still needs to be supported by necessary transformations, *i.e.*, patching, to convert the convolutional layers into generalized matrix multiplications [181]. The reliance on additional data preprocessing may call for attendant costs in electrical peripherals and block the overall calculation speed.

To tackle the problems, several works have focused on optical patching schemes (OPSs), which attempt to conduct the patching operation in a fully optical manner [176, 177]. In the prior phase, Bagherian et al*.* had managed to accomplish that by utilizing the so-called optical delay lines [182]. A more concise scheme based on this was later proposed by Xu et al*.* [176]. As depicted in Fig. 9f, they largely reduced the hardware cost by taking the advantage of the WDM technology, where a single optical delay line bank could be reused multiple times, and only one modulator is needed for each input channel. This optical CNN architecture, as simulated, could reach satisfactory performance (97% accuracy in MNIST) with a computational speed of more than 100 trillion MAC operations per second (TMAC/s), taking the practical hardware imperfections into account [177].

Currently, throughput and scalability are the main obstacles for WDM-MVM photonic processors. Emerging technologies like integrated comb lasers have created new prospects, which are capable to provide thousands of coherent laser lines at ultra-low energy costs [21]. With such support, an optical convolutional accelerator would achieve tremendous parallelism in a rather compact footprint. On this basis, Xu et al*.* [178] and Feldmann et al*.* originated the corresponding acceleration schemes from different perspectives in 2021. The multiplexing approaches have delivered remarkable results, though adopting one way alone is still far from exerting its performance limit. In this wise, Xu et al*.* conceived an approach to interleave time, wavelength, and space dimensions by utilizing the ubiquitous chromatic dispersion [171] in a manner similar to the effective pipeline architecture [183], as shown in Fig. 10a. More explicitly, the input 2D images are flattened into 1D vectors, then weighted via electro-optical modulation and fed into distinct wavelength channels, which are subsequently associated with progressive transmission delays. The following operations are piled up pixel by pixel in each “pipeline cycle”, *i.e.*, the symbol period, and the results of each given convolution window, or receptive field is simultaneously generated by a high-speed photoelectric detection per kernel [184]. In this way, an optical convolutional accelerator can reach a high efficiency of beyond ten trillion operations per second. Although ultra-low energy consumption is one of the fascinating advantages in optical computing, the weighting operations are mostly conducted in a volatile manner that requires a durative power supply to maintain the working condition [172, 173]. As shown in Fig. 10b, Feldmann *et.al* resolved this issue by combining two cutting-edge technologies to mitigate both latency and data movement, thereby cutting down the power dissipation. In this work, the soliton comb lasers provide massively parallel data transfer, and the phase change memories couple a fixed convolutional kernel to the interconnected waveguides in a non-volatile way. The designed photonic tensor core achieves an operating speed of 2 TMAC/s, which serves to be an encouraging prototype to exploit the in-memory computing paradigm. Nevertheless, there remain high demands for ultra-stable comb generation. Taking a step forward, reliable mass production, as well as the foresight of full commercialization could turn into tangible profits when further integrating lasers and microcombs in single wafers for the purpose of such applications [21].

Optical computing has great potential due to its ultra-fast propagation speed, ultra-high transmission bandwidth, and ability to accommodate multiple independent data channels. On the other hand, there is an essential requirement for light generation for an optical computing system. From simple operations like MVM to complicated computations like AI algorithms, light sources are necessary for providing the modulated input signals or performing the non-linear activation [185, 186]. While optical computing is particularly versed in massively parallel computing, practical applications are still under exploration, and new prospects are enlightened by evolving or emerging technologies. Looking forward, co-packaging the laser sources and other devices may endow optical computing with greater potential to take place in the field of high-performance information processing.

## Perspective and future outlook

**Fig. 11:**
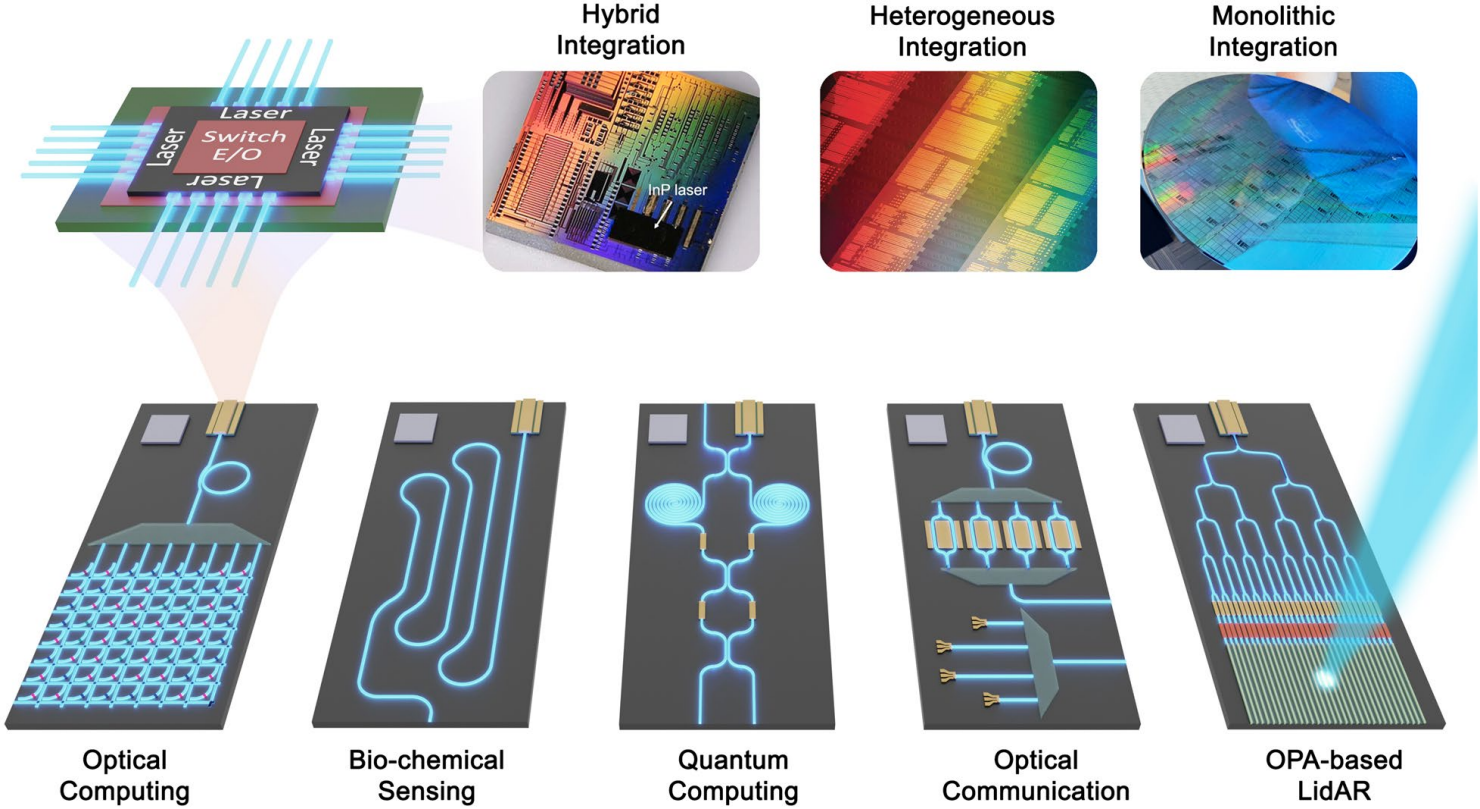
PICs in different system-level applications with integrated on-chip lasers via different integration techniques. Insets adapted with permission from [21, 189, 190], copyright 2016 & 2021, OPG & AAAS & NPG

At its conception, the vision of silicon photonics was met with a fair deal of skepticism, as silicon had been perceived as an “electrical” material rather than an “optical” material. This was a result of a few inherent drawbacks that plagued silicon, namely, being a highly-lossy medium towards high-power guided light, in addition to the inversion-symmetric nature of silicon crystals exhibiting a very weak Pockels effect and almost no second-order nonlinearities [187]. Hence, silicon has virtually zero electro-optic (EO) coefficients, thus making silicon-based optical modulators seem a far-fetched goal. More significantly, as have been discussed earlier, silicon on its own is a very inefficient light source, due to its indirect bandgap. In other words, at face value, silicon posed as a weak contender, be it as a light source, a modulator, a photodetector, etc. Yet, the call from the datacom and microelectronic industries was becoming louder and louder for more bandwidth and power-efficient solutions in transmitting data to substitute the over-exhausted copper-interconnected electronic computing ecosystem. As data rates increase from hundreds of Gbps to Tbps, it became paramount to reduce the associated transmission and switching power dissipation, and so, target power requirements have dropped over the years from nJ/bit to sub-pJ/bit [188]. Furthermore, the prospect of silicon photonics and silicon integrated circuits was undeniably too compelling, owing chiefly to their ability to use and be seamlessly-integrated with the already matured high-quality CMOS fabrication technology that can achieve high-volume yields at low die-costs, in addition to excellent uniformity and a large degree of scalability. All of that, thanks to an abundance of research and development from both academia and the industry, has culminated in multiple solutions over the years to surmount said shortcomings, and as time progressed, new approaches have been increasingly conceived. Indeed, myriad chip-level devices have matured today based on silicon photonic processes for various applications, as have been explored throughout this paper, incorporating on-chip lasers by the virtue of the different previously-discussed integration techniques, as illustrated in Fig. 11. However, the design and process routes of major manufacturers still vary to a large degree, and from this point of view, PIC technology is still in its infancy, where the ultimate solution with the optimal cost-performance-reliability balance has yet to stand out from the crowd.

As has been previously discussed, PICs are already commercialized and deployed in data centers in the form of optical interconnects on both inter- and intra-rack levels enabling scale-out computing systems as a part of the traditional data center infrastructures and ecosystems of pluggable optical transceivers. Be that as it may, the ultimate goal remains to implement silicon photonic devices on the chip itself in a co-packaged paradigm with integrated lasers to directly interface with the silicon chip itself.

**Fig. 12:**
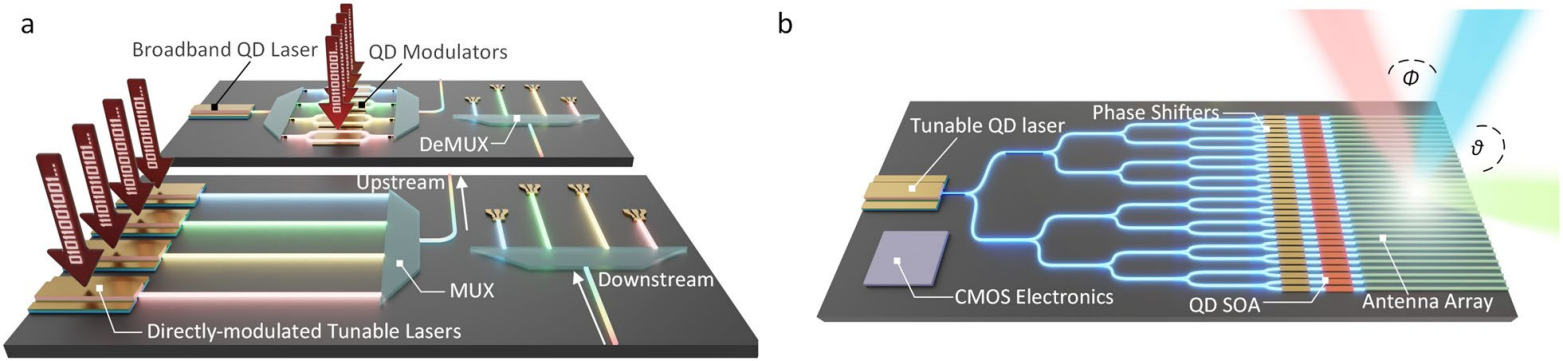
Integrated QD lasers for: **a** optical WDM communications and **b** OPA-based LiDAR applications

In contrast to the numerous integrated lasers required in the commercialized 200G, 400G (4 lasers), and even the future 800G (8 lasers) transceivers, which are deployed to realize the different parallel wavelength channels in CWDM architectures, a single microcomb source stands as a strong candidate to replace them and singularly provide the same number of wavelength channels, cutting down the cost, power, and physical footprint drastically. Such microcomb sources can and have been realized with the aid of microcomb high-*Q* resonators that are carefully tuned and designed to achieve soliton microcomb emissions. Alternatively, QD lasers can achieve the coveted microcomb emission with high-power flat-top spectra owing to their inherent inhomogeneously-broadened gain profiles. In addition to a suite of desirable features of QDs described previously, the near-zero linewidth enhancement factors make them promising for frequency and linewidth-sensitive applications, such as coherent-based communications (Fig. 12a) and OPA-based LiDAR (Fig. 12b).

In optical communications, QD lasers can be utilized as a directly-modulated tunable laser source in WDM architectures (bottom chip in Fig. 12a), as well as a microcomb source (top chip in Fig. 12a), where each of the wavelength-demultiplexed signals can be externally-modulated, potentially, by QD-based modulators. On the other hand, in OPA-based LiDAR systems, QDs can be employed as tunable sources and as the phase shifters in the form of EO modulators as well as gain elements in the form of SOAs, as illustrated in Fig. 12b, allowing for uniform, modular components. Furthermore, with proper design and tuning, QD lasers can be fabricated as passively mode-locked lasers, with ultrafast absorption recovery responses, that can generate ultra-short pulses (in the order of tens of femtoseconds) with very narrow beating RF linewidths [191], which can be utilized in time-division multiplexing (TDM). This set of properties makes integrated mode-locked QD lasers very promising not only as tunable or microcomb sources in WDM-based optical communications and interconnects, but also in LiDAR, biosensing, metrology, spectroscopy, and optical generation in millimeter wave (MMW) applications.

With that said, as a result of the homogeneous- and inhomogeneous-broadening phenomena in the active region of QD mediums, they typically suffer from high phase/frequency noise and, hence, broad optical linewidths (tens to hundreds of MHz), which is detrimental in many applications such as coherent transmission and LiDAR, etc. This is not to be confused with the very narrow RF linewidths associated with mode-locked QD lasers generated by beating consecutive emission pulses from the laser. Nonetheless, with the aid of self-injection-locking, *e.g.,* via MRRs, optical feedback can be exploited to suppress all the noisy emission components and lock them to match the main emission components, resulting in dramatically reducing the phase/frequency noise (70 dB demonstrated reduction) and, in turn, the optical linewidth by several orders of magnitude from the MHz-range to sub-kHz [192]. Strikingly, heterogeneously-integrated lasers on silicon have demonstrated even far narrower linewidths, compared to their monolithic III–V counterparts grown on native substrates. Although QD-based heterogeneously-integrated lasers with integrated resonators promise to meet the power efficiency and high-temperature operation concerns of various applications, their reliability yet needs further development to validate their implementation at a commercial level as a feasible solution. Furthermore, despite the fact that high-*Q* resonators can reduce the linewidth and be used for generating soliton microcomb emissions, they can result in undesirable non-linear effects that get more heightened inside the resonator due to their inherent intensity enhancement factor. One very promising contender to mitigate that is adopting the Si_3_N_4_ waveguide platform that can also enable better injection-locking, and hence lower phase noise and narrower linewidth, superior thermal stability, in addition to ultra-low optical losses [58, 61]. Another potential venture for development is multi-material integration beyond the traditional silicon-based material platforms, which has been and will likely continue to play a significant role in addressing different challenges.

On another front, machine learning-based AI and the increasing accessibility of high computational power can be leveraged in cross-layer optimization, design, and fabrication of different photonic components and systems, including on-chip integrated lasers, by relying on statistics and inference and learning from available heuristics and uncovering underlying hidden patterns. Furthermore, material informatics exploiting machine learning techniques, have the potential to reduce the number of growth runs and trials in MBE and CVD and to predict optimal growth conditions, especially in complex quantum structures, *e.g.,* heterogeneously- or monolithically- integrated QD structures, which have very stringent growth and strain management requirements. Machine learning can also be utilized in high-capacity large integrated systems in planning, performance prediction, system maintenance, fault prevention, and system resource allocation and management. Incorporating that, while also transitioning the optimization focus of on-chip lasers from individual device-level to application-specific system-level integration, bears major opportunities in the future, and optimization based on balancing between functionality-integration density and fabrication compatibility-complexity-yield is expected to continue to direct the industry towards the best interest for each specific application in the next decade.

As has been previously discussed, data transfer remains the largest bottleneck in terms of computing performance in the industry. Optical computing has the ability to compute data whilst still being transferred, cutting out switch delays, which will completely change how computer architectures are designed and perceived. When the computer industry moved from the vacuum tube to the transistor, latency decreased in magnitude of order from microseconds to nanoseconds. Photonics promises to reduce latency by orders of magnitude again, in the order of femtoseconds or even less. This speed factor alone would radically transform the computing industry. However, optical computing offers many other features as well. Generally, classical computers operate in serial pipelines, with each calculation being performed in cascade one after the other. In other words, in order to scale up to more complex problems, more processor cores are required, which translates to increased computing power requirements and more complex data management. Optical computers, on the other hand, can operate in parallel to tackle complex problems, due to wider bandwidths as compared to electronic-based computers, due to the ability to transport multiple wavelengths of light at the same time in WDM and other multiplexing configurations that have been discussed earlier. This can be enabled by leveraging the lessons and techniques used and discussed in integrated optical communication and interconnects from microcomb sources to mode-locked QD on-chip lasers assisted with self-injection locking. These factors of increased parallelism, power efficiency, and bandwidth translate to extremely scalable systems which are much more powerful, cost-effective, and do not suffer from severe complex data management issues. In addition, photonic-based computing will also play a significant role in quantum computing, due to the particle-wave duality of light. In the grand scope of things, fiber optic speeds will be slowed down no longer by bottlenecks imposed by traditional electronics and interconnects. From long-haul optical fiber-connected internet to integrated photonic interconnects, and then to all photonic computing, the long-term goal of computing at the speed of light enabled by an all-optical-framework will be finally within grasp, producing massive performance and efficiency gains in the future.

## Conclusion

In this paper, we have reviewed the state of the art and given our perspectives on the recent advancements in integrated on-chip lasers in photonic integrated circuits on a device-level of integration; and on system-level applications. At the device level, we explained the different approaches and efforts taken in integrating lasers in PIC chips, be they IV- or III–V-based lasers through different integrating methodologies while discussing the merits and associated obstacles of each route. Then, we highlighted the ongoing research in the most prominent system-level applications that can benefit greatly from PICs with on-chip lasers and show very promising prospects for development in optical communications and interconnects, to LiDAR, to bio-chemical sensing, to quantum and optical computing. This paper highlights the prospects of further development in incorporating PIC with on-chip lasers in system-wide applications to achieve more cost-effective, energy-efficient, high-performance solutions by leveraging the advantages of photonic integrated circuits and silicon photonics that have made them such an attractive platform in the first place.

## Data Availability

Not applicable.
